# Supplementary material for: Causal Relationship between Aging and Anorexia Nervosa: A White-Matter-Microstructure-Mediated Mendelian Randomization Analysis
Source: Biomedicines. 2024 Aug 16;12(8):1874. doi: 10.3390/biomedicines12081874 (PMC11351342; doi:10.3390/biomedicines12081874)
Supplement: Supplementary file 1 [file biomedicines-12-01874-s001.zip › Supplementary Figure/Figure S8/Leaveoneout plot of MR analysis for TL to ubm-b-2041.pdf]

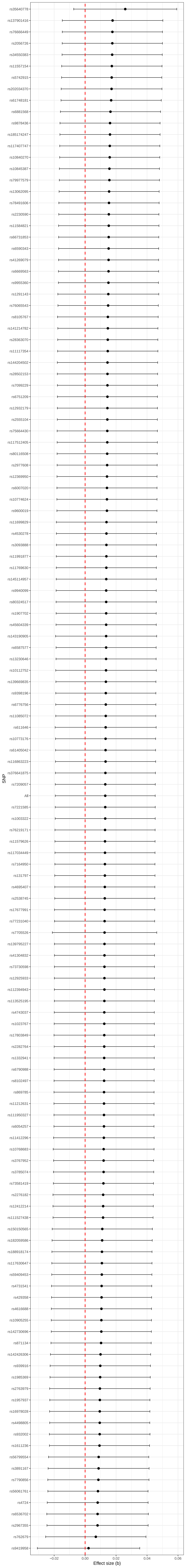
SNP
Effect size (b)
−0.02
0.00
0.02
0.04
0.06
rs35640778
rs137901416
rs76666449
rs2056726
rs34550383
rs11557154
rs5742915
rs202034370
rs61748181
rs6881568
rs9878436
rs185174247
rs117407747
rs10840270
rs10845387
rs79977579
rs13062095
rs78491606
rs2230590
rs11584821
rs66731853
rs6590343
rs41269079
rs6669563
rs9955360
rs1291143
rs76065543
rs8105767
rs141214782
rs28363070
rs11117354
rs144204502
rs28502153
rs7099229
rs6751209
rs12932179
rs2555104
rs75664430
rs117512405
rs80116508
rs2977608
rs12369950
rs6007020
rs10774624
rs9600019
rs11699829
rs4530278
rs3093888
rs11991877
rs11769630
rs145114957
rs9940099
rs80324517
rs1907702
rs45604339
rs143190905
rs6587577
rs13230646
rs10112752
rs139669835
rs9398196
rs6776756
rs11085072
rs611646
rs10773176
rs61405042
rs116863223
rs376641875
rs7209057
All
rs7221585
rs1003322
rs76219171
rs11579626
rs117034449
rs7164950
rs131797
rs4695407
rs2538745
rs17677991
rs77231040
rs7705526
rs139795227
rs41304832
rs73730598
rs12925933
rs112394943
rs113525195
rs4743037
rs1023767
rs17803849
rs2282764
rs1332941
rs6790988
rs8102497
rs869785
rs11212631
rs111950327
rs6054257
rs11412296
rs10768683
rs3767952
rs3785074
rs73581419
rs2276182
rs12412214
rs111527438
rs150150565
rs182059586
rs188918174
rs117630647
rs59409453
rs4731541
rs429358
rs4616688
rs10905255
rs142730696
rs871134
rs142426306
rs939916
rs1985369
rs2763979
rs1957937
rs16978028
rs4498805
rs932002
rs1611236
rs56799554
rs3891167
rs7790856
rs56061761
rs4724
rs6536702
rs2967355
rs762679
rs9419958
